# Metapristone (RU486-derivative) inhibits endometrial cancer cell progress through regulating miR-492/*Klf5*/*Nrf1* axis

**DOI:** 10.1186/s12935-020-01682-1

**Published:** 2021-01-07

**Authors:** Yue Chang, Min Hao, Ru Jia, Yihui Zhao, Yixuan Cai, Yun Liu

**Affiliations:** Department of Obstetrics and Gynecology, Beijing Friendship Hospital Affiliated to Capital Medical University, Beijing, China

**Keywords:** Endometrial cancer, miR-492, Metapristone, Klf5, Nrf1

## Abstract

**Background:**

Endometrial cancer is an invasive gynecological cancer prevalent in the world. The pathogenesis of endometrial cancer is related to multiple levels of regulation, referring to oestrogen, tumor-suppressor gene (e.g. *PTEN*) or microRNAs (e.g. miR-23a and miR-29b). Metapristone is a hormone-related drug, which is widely used in clinical treatment of endometrial cancer. However, the underlying regulatory mechanism of metapristone on endometrial cancer is still unclear, especially the regulatory effect on microRNAs. The aim of this study is to investigate the specific molecular mechanism of metapristone regulating microRNAs in the treatment of endometrial cancer.

**Methods:**

RL95-2 cells and Ishikawa cells were used as the endometrial cancer models. MiR-492 or si-miR-492 was transfected into RL95-2 cells and Ishikawa cells to explore the role of miR-492 in endometrial cancer. The cell cancer model and mice cancer model were used to confirm the function and mechanism of metapristone affected on endometrial cancer in vitro and in vivo. Mechanically, cell proliferation was monitored using MTT assay, cell colony formation assay and EdU assay. Luciferase reporter assay was used to identify the downstream target gene of miR-492. The protein expression and RNA expression were respectively measured by western blot and qRT-PCR for cell signaling pathway research, subsequently, were verified in the mice tumor model via immunohistochemistry.

**Results:**

Metapristone as a kind of hormone-related drug significantly inhibited the endometrial cancer cell growth through regulating cell apoptosis-related gene expression. Mechanically, miR-492 and its target genes *Klf5* and *Nrf1* were highly expressed in the endometrial cancer cell lines, which promoted cell proliferation and inhibited cell apoptosis. Metapristone decreased the expression of miR-492 and its target genes *Klf5* and *Nrf1*, leading to endometrial cancer cell growth inhibition in vitro and in vivo.

**Conclusion:**

Metapristone inhibited the endometrial cancer cell growth through regulating the cell apoptosis-related signaling pathway and decreasing the expression of miR-492 and its downstream target genes (*Klf5* and *Nrf1*), which provided the theoretical basis in clinical treatment of endometrial cancer.

## Introduction

Endometrial cancer is one of the most aggressive cancers in female, after breast cancers, lung cancers, and colorectal cancers (CRCs) [1]. In female-specific cancers, the mortality of endometrial cancer is much higher than others, including ovarian and cervical cancers [2]. According to the pathological features, endometrial cancer is classified into two types: Type I is the most common endometrioid adenocarcinoma (80–90%) and Type II includes non-endometrioid subtypes such as serous, clear cell and undifferentiated carcinomas, as well as carcinosarcoma/malignant-mixed Müllerian tumor (10–20%) [3]. Numerous risk factors can bring about endometrial cancer. One type of risk factors is bound up with hormone. Unopposed exposure of the endometrium to estrogen is the main risk factor for type I endometrial cancer, including polycystic ovary syndrome (PCOS) [4]. Other factors including family, old age, thyroid disease, hypertension, Lynch syndrome, diabetes mellitus and obesity also increase the risk of endometrial cancer [1, 5]. From molecular viewpoint, endometrial cancer resembles proliferative rather than secretory endometrium, which suggests that the abnormity of tumor-suppressor genes such as *PTEN*, *KRAS*, *TP53* and *ERBB-2* can accelerate the development of endometrial cancer [6–10].

In recent decades, the research has penetrated into the various levels of the regulation of cancer-related genes. MicroRNAs (miRNAs) are post-transcriptional regulators that play roles of translational repression and gene silencing, which are involved in many essential biological processes, including cell proliferation, apoptosis, and differentiation. A great deal of studies have reported that miRNAs are correlative with multiple diseases such as inflammation and cancer [11]. Predictably, miRNAs have also participated in the pathogenesis and development process of endometrial cancer. MiR-23a inhibited endometrial cancer by targeting *sine oculis* homeobox homolog 1 (SIX1) [12]. MiR-29b played important roles in proliferation and progression of endometrial cancer cells by direct regulation of *PTEN *[13]. However, the effect of miRNAs on endometrial cancer deserves to be explored in greater depth to find the more efficient clinic treatment options.

In China, many patients are diagnosed with advanced stage of endometrial cancer and have rapid progress of disease in 1 year [14]. To date, surgery remains the primary option for the early-stage patients. Chemotherapy is appropriate for advanced and recurrent patients, which has some side effects. However, tumor heterogeneity and drug resistance are major obstacles to chemotherapy for endometrial cancer, resulting in the limitation of chemotherapy benefits to a subgroup of patients [15, 16]. Therefore, it is urgent to find a new kind of drug with better curative effects and less side effects for endometrial cancer clinical treatment.

Estrogen is one of crucial hormone in human especially female and it exhibits a vital and broad spectrum of physiological functions, including developing and maintaining both the reproductive system and menstrual cycle, modulating the bone density, regulating the brain function and cholesterol mobilization. Nevertheless, estrogen also contributes to pathological complications especially breast cancer and endometrial cancer. After years of investigation, the estrogen through estrogen receptor (ER) has been taken as a classical factor for endometrial cancer. The disorder of estrogen can lead to the tumorigenesis and development of endometrial cancer [1]. High expressed ER in endometrial cancer is usually thought to be driven by estrogen. Mostly, the ER of cell surface is activated after binding to estrogens, subsequently, initiates associated signaling pathways such as MAPK pathway [17] and Akt pathway [18], to regulate cell proliferation and cell apoptosis. Considering the above, synthetic progestin is main course of treatment for type I endometrial cancer. The progesterone receptor (PR) is activated and blocks the pro-growth actions of ER in cell autonomous fashion by regulating the related genes expression through binding the similar targeting sites. Progestin is generally used in combination with tamoxifen in cycling period to enhance the function of PR. Hence, drugs related with estrogen regulation have tremendous potential to treat endometrial cancer [19–21].

RU486 (mifepristone) is a derivative of synthetic norethindrone and exhibits the activity of anti-progesterone and anti-glucocorticoid through blocking PR, androgen receptor (AR) and glucocorticoid receptor (GR) [22, 23]. RU486 has been developed as a kind of effective chemopreventive agent against cancer metastasis and presents antitumor function that has been assessed in various cancer cell lines, mice models and clinical trials [24]. While the exact mechanisms, associated signaling pathways and targets of RU486 towards endometrial cancer are poorly understood. Similarly, metapristone (RU42633) is the primary metabolite of RU486 [25], which is used to terminate pregnancy in the first month in clinic [26]. Interestingly, metapristone could inhibit some kinds of cancer cell proliferation [27, 28]. However, few reports indicated that whether metapristone could treat endometrial cancer. Consequently, it is of great significance for clinical treatment to explore the role of metapristone on endometrial cancer.

In this study, we chose the hormone-related inhibitor metapristone to treat endometrial cancer. The results indicated that miR-492 was highly expressed in endometrial cancer cell lines. The study aimed to find out the role of miR-492 in the development of endometrial cancer in vitro and in vivo, and the molecular mechanism of therapeutic effect of metapristone on endometrial cancer, which provided the theoretical basis for clinical treatment of endometrial cancer.

## Materials and methods

### Cell culture

Cancer cell lines, including RL95-2 cells (ATCC^®^ CRL-1671), Ishikawa cells (ECACC, 99040201), MCF7 cells (ATCC^®^ HTB-22), BT-474 cells (ATCC^®^ HTB-20), A549 cells (ATCC^®^ CCL-185) and MGC-803 cells (BLUEFBIO, BFN608007257) were obtained from American Type Culture Collection (Manassas, VA, USA) and European Collection of Authenticated Cell Cultures (Porton Down, UK). These cells were cultured with Dulbecco’s modified Eagle’s medium (DMEM) which contained 1% penicillin–streptomycin solution (Gibco, USA) and 10% FBS. The cultured condition was at 37˚C in 5% CO2 humidified atmosphere (HERAcell 150i/240i, Thermo, USA). STR profiling and mycoplasma contamination were performed to keep the authenticity of cell line on regular basis.

### Transfection

The sequence of miR-492 was obtained from National Center for Biotechnology Information (NCBI). The small interfering RNA (siRNA) targeting miR-492 (si-miR-492) and negative control (si-NC), miR-492 mimic and negative control (miR-NC) were purchased from Genepharma (Shanghai, China). Thereafter, these plasmids were transfected into RL95-2 cells and Ishikawa cells in the concentration of 100 nM for 48 h by use of Lipofectamine 2000 (Invitrogen, Carlsbad, CA). The sequences were shown in Additional Table 1.

### MTT assay

For the cell proliferation assay, the total of 2000 RL95-2 cells or Ishikawa cells were plated into 96-well plates each well. The cells were treated with ethanol or metapristone (50 μM), transfected with miR-NC or miR-492 mimic (100 nM), or transfected with si-NC or si-miR-492 (100 nM) for 5 days. The blank controls were the non-cell wells with medium. The culture medium was removed and 20 μl 3-(4,5-dimethylthiazol-2-yl)-2,5-diphenyltetrazolium bromide (MTT) (5 mg/ml) was added to each well for 4 h at 37 °C. Therewith, 150 μl of DMSO was added for 15 min. Cell numbers were counted at a wavelength of 570 nm by Model 680 Microplate Reader (Bio-Rad Laboratories, USA). The six replicates were in each treatment and three times were repeated for MTT assay [29].

### Cell colony formation assay

A total of 800 RL95-2 cells or Ishikawa cells were plated to 6-well plates with soft agar each well with the treatment of ethanol or metapristone (50 μM), transfected with miR-NC or miR-492 mimic (100 nM), or transfected with si-NC or si-miR-492 (100 nM). Two weeks later, cells were fixed with formaldehyde after being washed with phosphate-buffered saline (PBS) three times, and stained with Giemsa staining solution for 30 min at room temperature. Visible clones were counted by an inverted microscope (Olympus, Japan) [30]. Each experiment was repeated three times.

### EdU assay

A total of 5000 RL95-2 cells or Ishikawa cells were plated to 6-well plates each well and were treated with ethanol or metapristone (50 μM, 48 h), transfected with miR-NC or miR-492 mimic (100 nM, 48 h), or transfected with si-NC or si-miR-492 (100 nM, 48 h). EdU staining proliferation kit was purchased from Abcam (ab219801). The plates were added with EdU solution and were incubated for 3 h and then treated with 4% formaldehyde. After the process, the cells were stained with DAPI and performed as the instruction described by inverted microscope (Olympus, Japan). Each experiment was repeated three times.

### Luciferase reporter assay

Luciferase reporter assay was performed as the papers described [30, 31]. In brief, the wild type or mutanted 3′-UTR sequence of Klf5 and Nrf1 were cloned into pGL3-luc vectors (Promega, USA). A total of 3 × 10^5^ RL95-2 cells or Ishikawa cells were plated in 24-well plates 24 h prior to transfection. For each well, the reporter constructs (500 ng) and the miR-NC or miR-492 mimic (100 nM) were co-transfected into RL95-2 cells or Ishikawa cells by using Lipofectamine 2000. After transfection for 48 h, the cells were lysed and the relative luciferase activity was measured by using dual-luciferase reporter assay system (Promega, USA). The miR-492 reporter activity was normalized to empty vector control. Each experiment was repeated three times.

### Western blot

The RIPA buffer (Beyotime, China) was used to lyse all the cell samples and tumor tissues and total protein amounts were determined by BCA protein assay (Pierce Biotechnology, USA) and then denatured with Laemmli buffer (Sigma, USA) at 95 °C for 10 min. Total protein (30 mg) was separated by 10%-12% sodium dodecyl sulfate–polyacrylamide gel electrophoresis and was transferred to nitrocellulose membranes (Millipore, USA). Membranes were blocked at room temperature for 1 h with 5% non-fat milk in Tris-buffered saline with Tween 20 (TBS-T). Then, the membranes with antibodies were incubated at 4 °C overnight. The primary antibodies were as followed: Cleaved-Caspase-3 (dilution, 1:1000; Cell Signaling Technology, 9654), Cleaved-Caspase-9 (dilution, 1:1000, Cell Signaling Technology, 20750), Bax (dilution, 1:1000, Cell Signaling Technology, 2774), Bcl-2 (dilution, 1:1000, Cell Signaling Technology, 15071), Klf5 (dilution, 1:1000, Cell Signaling Technology, 51586), Nrf1 (dilution, 1:1000; Santa Cruz, sc-28379) and GAPDH (dilution, 1:1000; easybio, BE0023). Horseradish peroxidase-conjugated secondary antibodies were as followed: rabbit-HRP (easybio, 1:20,000; BE0101-100). The results were detected by ECL Western Blotting Substrate (Pierce, Thermo Fisher, USA) and captured with the ImageQuant LAS 400 imaging system (GE Healthcare Life Sciences, USA). Each experiment was repeated three times.

### Quantitative real-time polymerase chain reaction (qRT-PCR)

The total RNA of cells was extracted with Trizol (Invitrogen, USA), and then had the process of reverse transcription to cDNA Reverse Transcriptase M-MLV (TakaRa, Japan). QRT-PCR was performed with SYBR Green PCR reagents (CoWin Biosciences, China) on a Real-Time PCR System (Carlsbad, USA), and analyzed with StepOne Software (Thermo Fisher, USA). The expression of mRNA was normalized to GAPDH [30]. The primers were performed in Additional Table 1. The three replicates were in each treatment and three times were repeated for qRT-PCR.

### Drug administration

Mifepristone was purchased from Shanghai New Hualian pharmaceutical Co., with purity > 98%. Metapristone was synthesized, using mifepristone as the starting material as described previously [29]. Metapristone was dissolved in ethanol, so that ethanol was used as negative control.

### Immunohistochemistry

The tissues were fixed with formalin and embedded with paraffin. The tissue sections were divided into 5 μm thickness and the dehydration was reached with the different concentration ethanol after being dewaxed in xylene. The sections were oven heating in 0.01 M sodium citrate buffer (pH 6.0, Beyotime China) for 10 min to antigen retrieval. Hydrogen peroxide (3%) and BSA (5%) (Amresco, USA) were used to block endogenous peroxidase activity and nonspecific staining for 20 min and 1 h, respectively. The primary antibody of anti-Ki-67 (1:1000, Cell Signaling Technology, 9449), anti-Cleaved-caspase 3 (1:1000, Cell Signaling Technology, 9661), anti-Klf5 (1:1000, Abcam, ab137676) and anti-Nrf1 (1:1000, Santa Cruz, sc-28379) were incubated with tissue sections for overnight in 4 °C, then, were incubated with secondary antibodies for 1 h at room temperature. The cultured sections were incubated with streptavidin peroxidase for 10 min and 3,3-diaminobenzidine tetrachloride (DAB, TIANGEN China) was used to detect peroxidase activity [32].

### Tumor xenograft model

Female Balb/c nude mice (20 g, 7–8 weeks) were obtained from Vital River Laboratory Animal Technology (Beijing, China) and were kept in standard environment with the unified breeding. Mice were sacrificed by carbon dioxide asphyxiation. All the experiments were approved by the Animal Research Committee of the Beijing Friendship Hospital Affiliated to Capital Medical University and conformed to the ethical standards with the guidelines of the National Animal Care and Ethics Institution. Two million of RL95-2 cells or Ishikawa cells were diluted in 0.1 ml of saline and were injected subcutaneously into 7-weeks old female Balb/c nude mice. After the injection of cancer cells, the experimental group mice (more than 6 mice per trial) were intraperitoneally injected with metapristone (45 mg/kg, three times for one week) during two weeks and the control group mice were intraperitoneally injected with vehicle at the same time points. Tumor xenografts were measured using a caliper during the experiment. The tumor volume was calculated by this formula: volume = (length × width^2^)/2. After the sacrifice of mice, the tumors were separated for future analysis. Each group contained more than 6 mice [33].

### Statistical analysis

For all studies, statistical analyses were conducted using the GraphPad Prism software. All the results were analyzed with Mean ± SD. The significance analysis of all the experiments was used by student’s two-tailed non-paired t-test. P < 0.05 was considered to have statistical significance. *P < 0.05, **P < 0.01, ***P < 0.001, ****P < 0.0001.

## Results

### Metapristone inhibited endometrial cancer cell growth and promoted endometrial cancer cell apoptosis

**Fig. 1:**
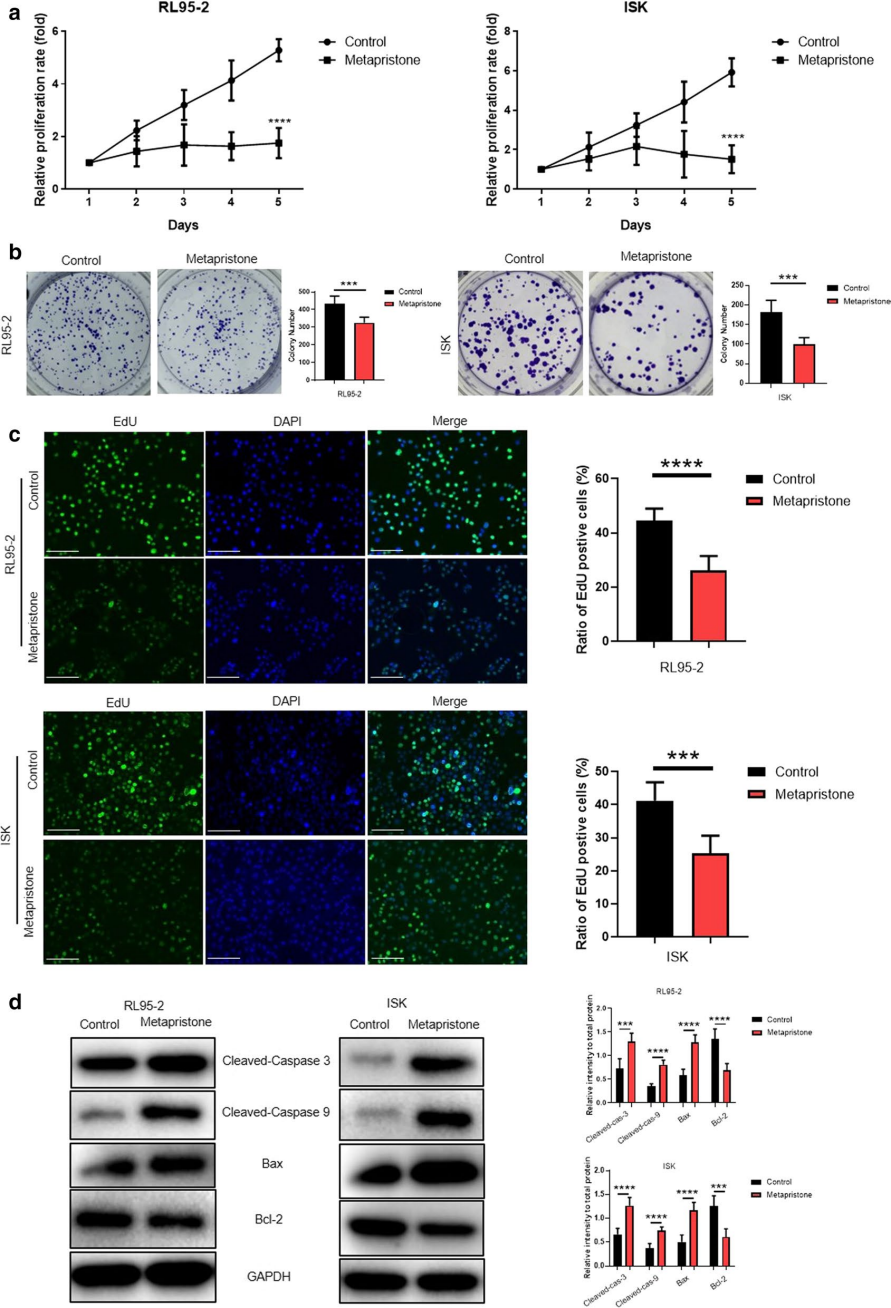
Metapristone inhibited endometrial cancer cell growth and promoted endometrial cancer cell apoptosis. **a** Cell growth curves by MTT assay of RL95-2 cells and Ishikawa cells with metapristone (50 μM) treatment. Ethanol-treated cells were as control. Results represent mean ± SD (n = 6). **b** Cell colony formation of RL95-2 cells and Ishikawa cells with metapristone (50 μM) treatment. Ethanol-treated cells were as control. Values are mean ± SD (n = 3 per group). **c** EdU assay was used to detect the cell proliferation phenotype with the treatment of ethanol or metapristone (50 μM 48 h). **d** The protein level of Cleaved-Caspase 3, Cleaved-Caspase 9, Bax and Bcl-2 in RL95-2 cells and Ishikawa cells with ethanol or metapristone treatment were detected by western blot. GAPDH was used as the control

Firstly, IC50 values of metapristone were observed in different cancer cells and the results demonstrated that RL95-2 cells and Ishikawa cells were more susceptible to metapristone (Additional Fig. 1). RL95-2 cells and Ishikawa cells as endometrial cancer cell models were used for future investigation. The cell growth curve results showed that the cell growth rate of RL95-2 cells and Ishikawa cells were obviously delayed with the treatment of metapristone (Fig. 1a). Analogously, the cell colony numbers were highly reduced when RL95-2 cells and Ishikawa cells were treated with metapristone (Fig. 1b). Moreover, compared to the control group, the positive staining cell numbers of metapristone-treated RL95-2 cells and Ishikawa cells in EdU assay were also significantly decreased (Fig. 1c). The above results suggested that metapristone could inhibit the endometrial cancer cell growth and proliferation. On the other hand, in order to further explore whether metapristone affected the cell apoptosis-related signaling pathways, the protein expression of some cell apoptosis-related genes was detected through western blot. As shown in Fig. 1d, metapristone increased the expression of cleaved-caspase 3, cleaved-caspase 9, Bax and decreased the expression of Bcl-2, which demonstrated that metapristone activated cell apoptosis-related signaling pathways. All these results indicated that metapristone inhibited endometrial cancer cell growth and proliferation, and accelerated endometrial cancer cell apoptosis.

### MiR-492 played the role of cell proliferation promotion and cell apoptosis resistance in endometrial cancer cells

**Fig. 2:**
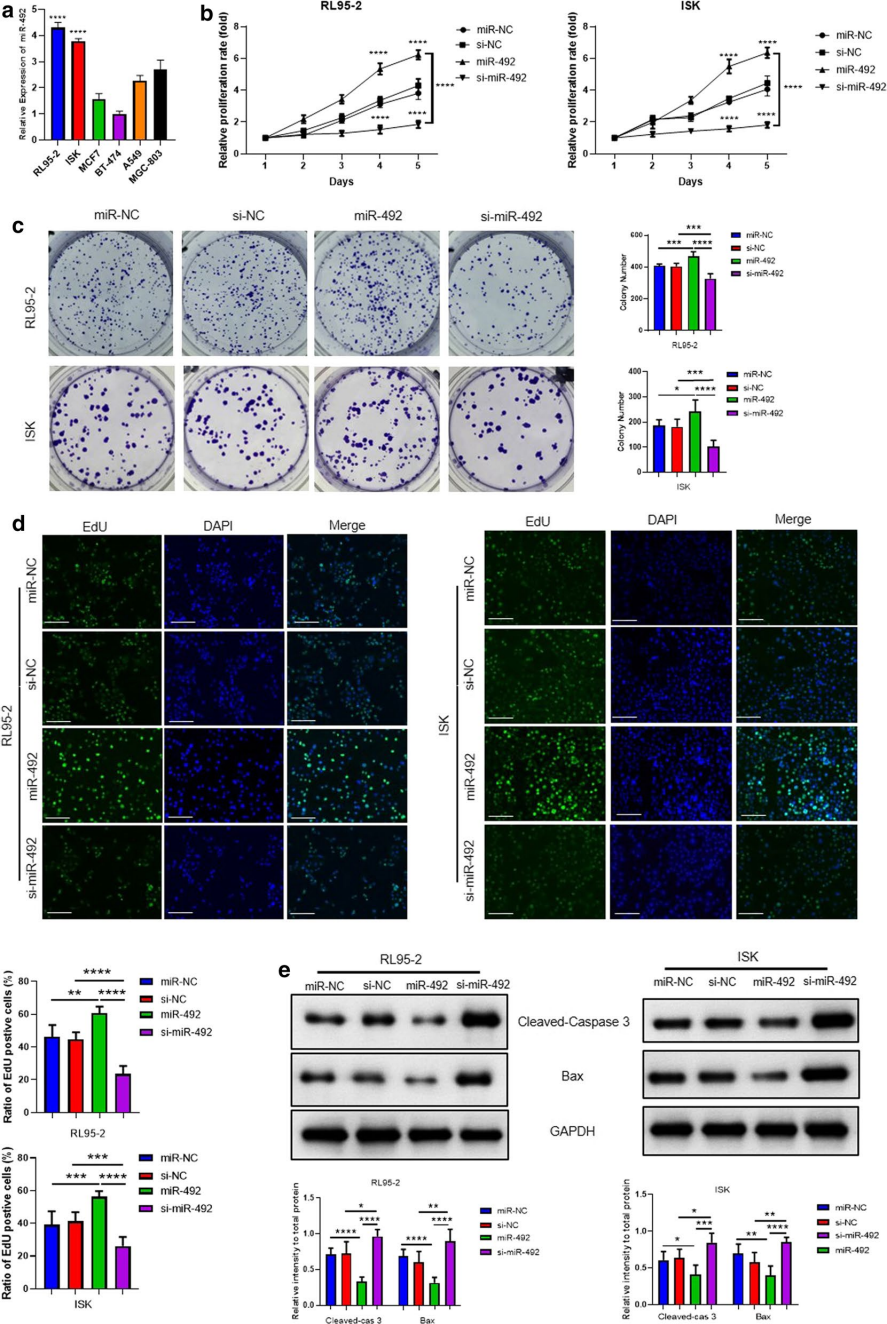
MiR-492 played the role of cell proliferation promotion and cell apoptosis resistance in endometrial cancer cells. **a** The mRNA expression of miR-492 of RL95-2, Ishikawa, MCF7, BT-474, A549 and MGC-803 cells were measured with real-time PCR. Values are means ± SD (n = 3). **b** Cell growth curves by MTT assay of RL95-2 cells and Ishikawa cells with miR-NC or miR-492 (100 nM) and si-NC or si-miR-492 (100 nM) treatment. Results represent mean ± SD (n = 3 per group). **c** Cell colony formation of RL95-2 cells and Ishikawa cells with miR-NC or miR-492 (100 nM) and si-NC or si-miR-492 (100 nM) treatment. Values are mean ± SD (n = 3). **d** EdU assay was used to detect the cell proliferation phenotype with miR-NC or miR-492 (100 nM) and si-NC or si-miR-492 (100 nM) treatment for 48 h. **e** The protein level of Cleaved-Caspase 3, Cleaved-Caspase 9, Bax and Bcl-2 in RL95-2 cells and Ishikawa cells with miR-NC or miR-492 (100 nM) and si-NC or si-miR-492 (100 nM) treatment for 48 h were detected by western blot. GAPDH was used as the internal control

Different types of cancer cell lines were chosen to detect the expression of miR-492. Interestingly, miR-492 was relatively highly-expressed in endometrial cancer cell lines RL95-2 cells and Ishikawa cells than other cancer cell lines, including breast cancer cell lines MCF7 cells and BT-474 cells, lung cancer cell line A549 cells and stomach cancer cell line MGC-803 cells (Fig. 2a). To better investigate the role of miR-492 in the development of endometrial cancer, the overexpression or knockdown experiments of miR-492 were designed. The RL95-2 cells and Ishikawa cells were transfected with miR-NC or miR-492, si-NC or si-miR-492, respectively. Interestingly, overexpression of miR-492 significantly expedited the endometrial cancer cell growth, on the contrary, knockdown of miR-492 retarded the endometrial cancer cell growth (Fig. 2b). Predictably, the results of cell colony formation assay resembled the results of cell growth curve. In comparation with control groups, the RL95-2 cells and Ishikawa cells with the transfection of miR-492 formed more cell clones and the opposite results were observed in the groups with the transfection of si-miR-492 (Fig. 2c). The EdU assay results also indicated that miR-492 promoted the cell proliferation of RL95-2 cells and Ishikawa cells and the treatment of si-miR-492 in the RL95-2 cells and Ishikawa cells were provided with the cell proliferation inhibition effect (Fig. 2d). In addition, the western blot results showed that miR-492 decreased the expression of cleaved-caspase 3 and pro-apoptotic protein Bax, which suggested that overexpression of miR-492 could inhibit the cell apoptosis. On the other hand, the knockdown of miR-492 revealed the completely different expression level of cell apoptosis-related protein with higher expression of cleaved-caspase 3 and Bax. All these results proved the role of miR-492 in cell proliferation promotion of endometrial cancer cells (Fig. 2e).

### *Klf5* and *Nrf1* were downstream target genes of miR-492

**Fig. 3:**
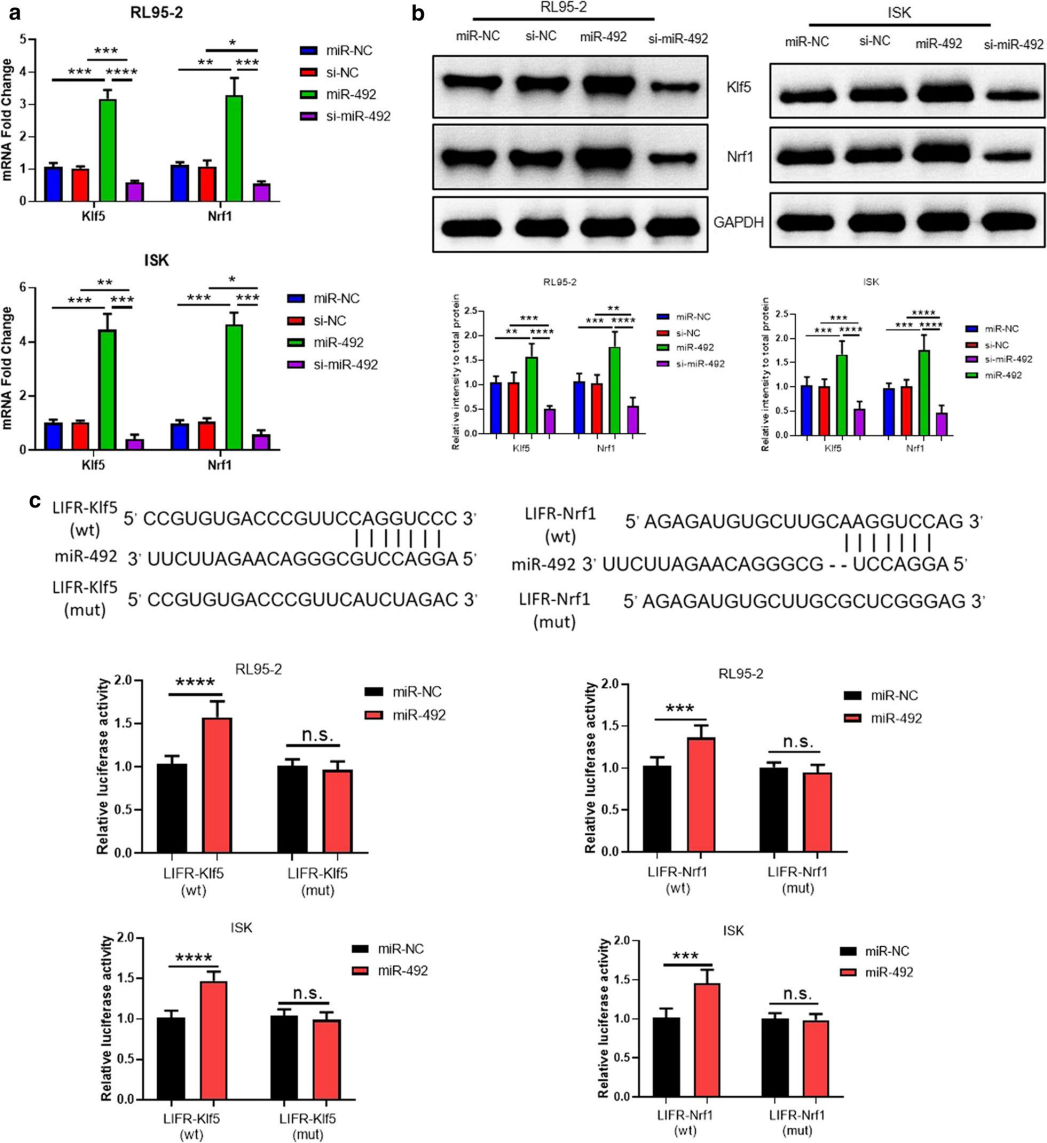
*Klf5* and *Nrf1* were downstream target genes of miR-492. **a** The mRNA level of *Klf5* and *Nrf1* in RL95-2 cells and Ishikawa cells with miR-NC or miR-492 (100 nM) and si-NC or si-miR-492 (100 nM) treatment for 48 h were measured by real-time PCR. Values are means ± SD (n = 3). **b** The protein level of *Klf5* and *Nrf1* in RL95-2 cells and Ishikawa cells with miR-NC or miR-492 (100 nM) and si-NC or si-miR-492 (100 nM) treatment for 48 h were detected by western blot (n = 3 per group). Values are means ± SD. **c** Prediction of the binding sites for miR-492 in wild-type (wt) of *Klf5* and *Nrf1*. The mutated (mut) LIFR-Klf5 and mutated (mut) LIFR-Nrf1 showed the disruption of miR-492 binding sites in RL95-2 cells and ISK cells. Values are means ± SD

To further study the specific molecular mechanism of miR-492 effect on the endometrial cancer cells, the potential downstream target genes of miR-492 were predicted online. The results suggested two latent target genes, *Klf5* and *Nrf1*, both of which were transcription factors. Subsequently, the mRNA expression of *Klf5* and *Nrf1* were detected through qRT-PCR when the RL95-2 cells and Ishikawa cells were transfected with miR-NC or miR-492, si-NC or si-miR-492, respectively. The results showed that the mRNA expression of *Klf5* and *Nrf1* were obviously increased when miR-492 was overexpressed and were decreased with the treatment of si-miR-492 in the RL95-2 cells and Ishikawa cells (Fig. 3a). As Fig. 3b shown, miR-492 also enhanced the protein expression of *Klf5* and *Nrf1*, and the protein expression of *Klf5* and *Nrf1* were weakened in the knockdown experiments of miR-492. All these data indicated that *Klf5* and *Nrf1* were potential target genes of miR-492. Furthermore, the luciferase reporter assay was designed to directly proved that *Klf5* and *Nrf1* were downstream target genes of miR-492. Wildtype *Klf5* or *Nrf1* promoter complementary toward miR-492 reinforced miR-492-induced promoter activity, whereas mutations of both *Klf5* and *Nrf1* promoter shown in Fig. 3c did not influence the miR-492-induced promoter activity (Fig. 3c). Collectively, all these results suggested that *Klf5* and *Nrf1* were direct downstream target genes of miR-492.

### Exogenous overexpression of miR-492 resisted the metapristone-induced antitumor effect in endometrial cancer

**Fig. 4:**
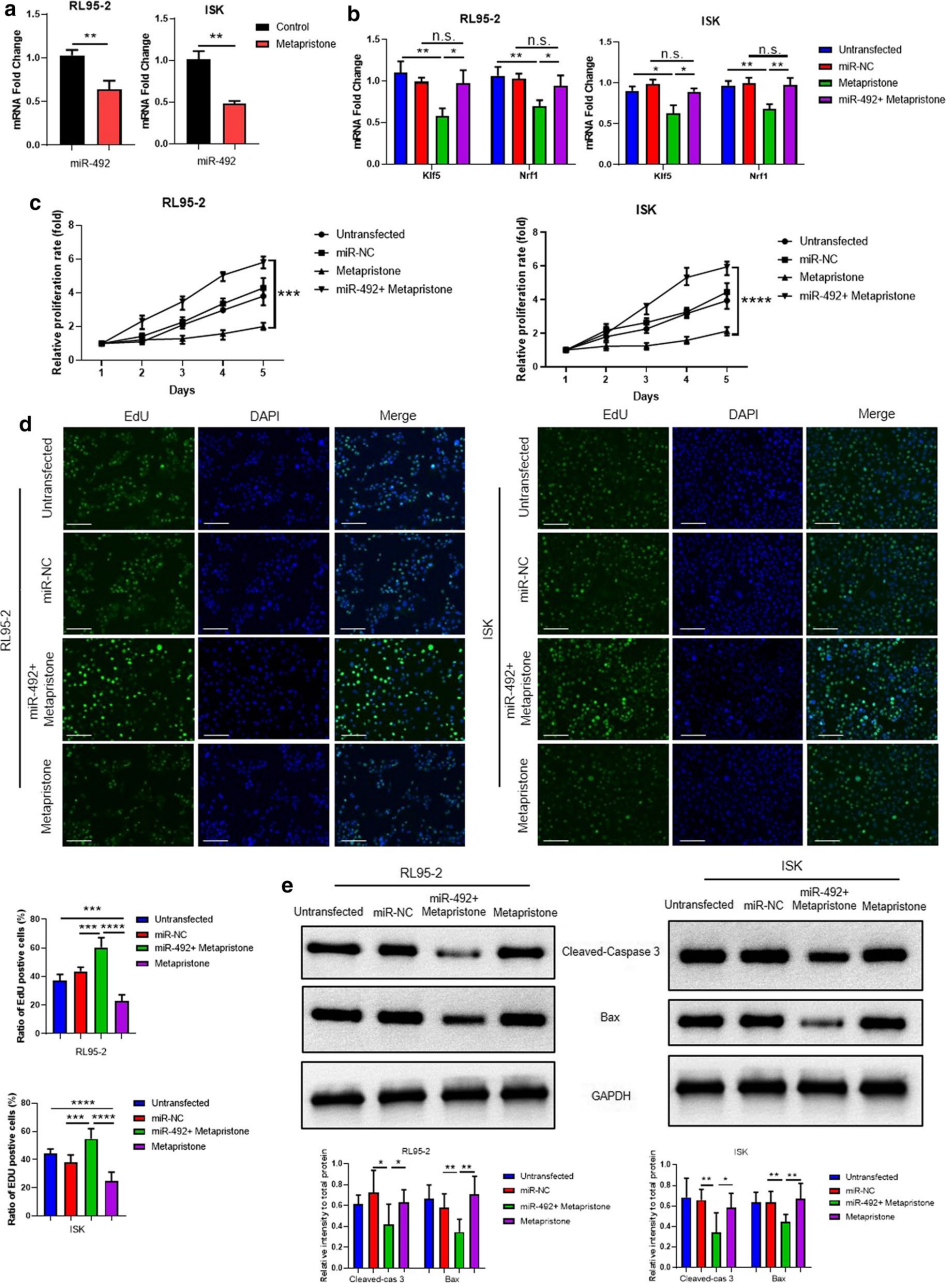
Exogenous overexpression of miR-492 resisted the metapristone-induced antitumor effect in endometrial cancer. **a** The mRNA expression of miR-492 of RL95-2 cells and Ishikawa cells with ethanol or metapristone treatment (50 μM 48 h) were measured with real-time PCR. Values are means ± SD (n = 3 per group). **b** The mRNA expression of *Klf5* and *Nrf1* in RL95-2 cells and Ishikawa cells with or without metapristone and miR-492 + metapristone treatment (50 μM 48 h) were detected by real-time PCR. Non-treated and mir expression system cells were as control. Values are means ± SD (n = 3 per group). **c** Cell growth curves by MTT assay of RL95-2 cells and Ishikawa cells with or without metapristone and miR-492 + metapristone treatment (50 μM 48 h). Non-treated and mir expression system cells were as control. Results represent mean ± SD (n = 6 per group). **d** EdU assay was used to detect the cell proliferation phenotype with or without metapristone and miR-492 + metapristone treatment (50 μM 48 h) in RL95-2 cells and Ishikawa cells. Non-treated and mir expression system cells were as control. Results represent mean ± SD (n = 6 per group). **e** The protein expression of Cleaved-caspase 3 and Bax in RL95-2 cells and Ishikawa cells with or without metapristone and miR-492 + metapristone treatment (50 μM 48 h) were detected by western blot. Values are means ± SD. Non-treated and mir expression system cells were as control (n = 6 per group)

To further clear the mechanism of metapristone effect on endometrial cancer cell whether through miR-492, the mRNA expression of miR-492 and its downstream target genes *Klf5* and *Nrf1* were detected in metapristone-treated RL95-2 cells and Ishikawa cells. The results showed that metapristone substantially reduced the expression of miR-492 (Fig. 4a) and its downstream target genes *Klf5* and *Nrf1* (Fig. 4b). Interestingly, exogenous overexpressed miR-492 restored the mRNA expression of *Klf5* and *Nrf1* that were down-regulated with the treatment of metapristone (Fig. 4b). So, the effect on endometrial cancer cell growth, proliferation and apoptosis with the co-administration of both metapristone and miR-492 aroused our great interests. The results of Fig. 4c showed that RL95-2 cells and Ishikawa cells had faster growth rate with administration of both miR-492 and metapristone, even more than the control groups. Similarly, the EdU assay results indicated that the combination of miR-492 and metapristone exhibited the strongest cell proliferation promotion effect, which was totally different from the single treatment of metapristone (Fig. 4d). All the above results suggested that miR-492 restored the metapristone-mediated cell proliferation inhibition. Moreover, the cell apoptosis-related signaling pathways were also detected via western blot. The results demonstrated that the co-administration of miR-492 and metapristone decreased the protein expression of cleaved-caspase 3 and Bax (Fig. 4e), which suggested miR-492 restored the cell apoptosis that was induced by metapristone. All these results proved that metapristone inhibited the expression of miR-492 and its downstream target genes *Klf5* and *Nrf1*. Exogenous overexpression of miR-492 resisted the antitumor effect of metapristone through affecting the cell growth, proliferation and apoptosis.

### Metapristone inhibited the tumor growth through regulating miR-492 and its downstream target genes Klf5 and Nrf1 in vivo

**Fig. 5:**
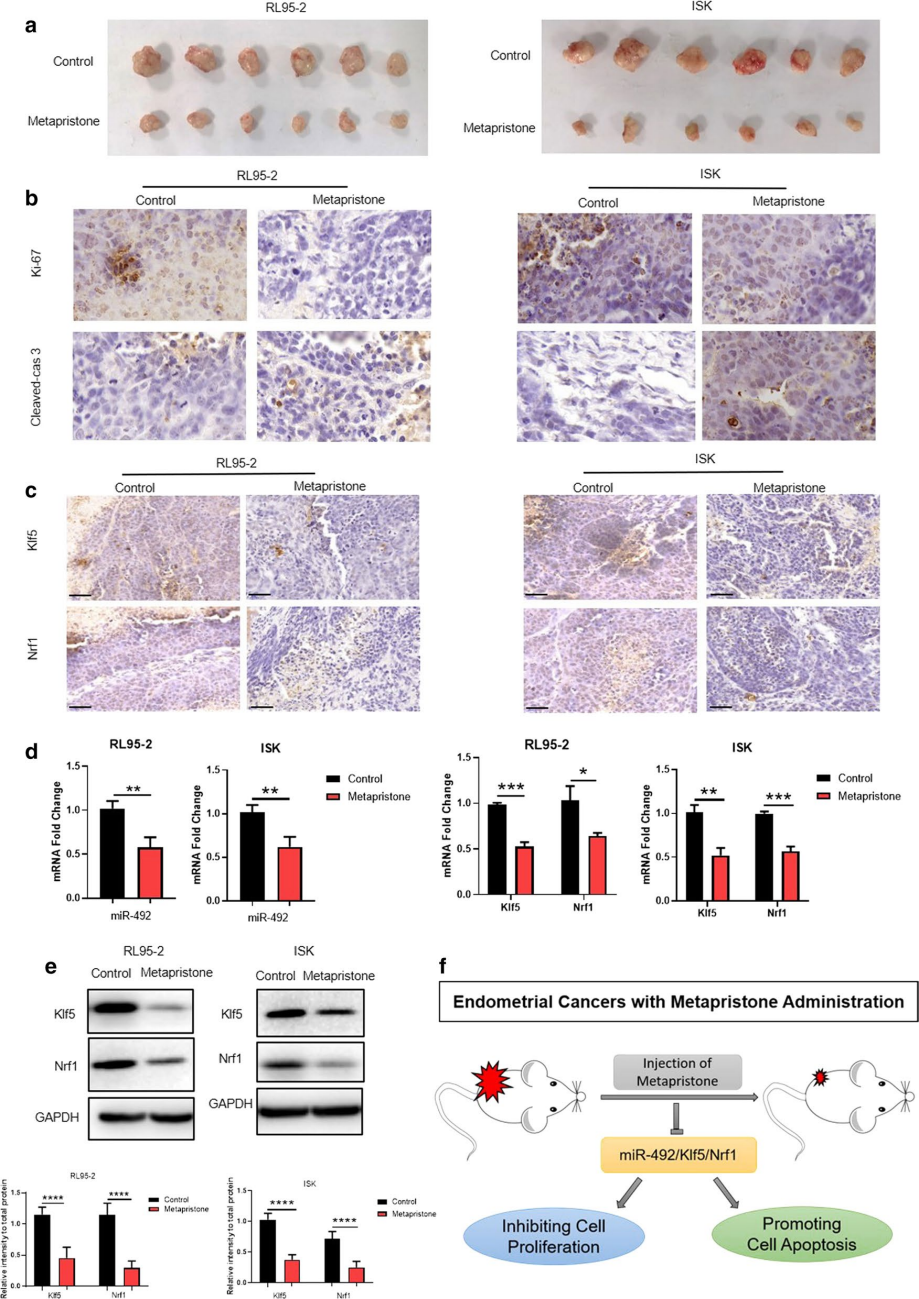
Metapristone inhibited the tumor growth through regulating miR-492 and its downstream target genes Klf5 and Nrf1 in vivo. **a** The tumor xenograft model of RL95-2 cells and Ishikawa cells. (n = 6 per group). **b** The expression of Ki-67 and Cleaved-caspase 3 in the tumor xenograft of RL95-2 cells and Ishikawa cells with or without metapristone treatment were detected by immunohistochemistry. Magnification: × 200. **c** The expression of *Klf5* and *Nrf1* in the tumor xenograft of RL95-2 cells and Ishikawa cells with or without metapristone treatment were detected by immunohistochemistry. Magnification: × 50. **d** The mRNA expression of miR-492, *Klf5* and *Nrf1* in the tumor xenograft of RL95-2 cells and Ishikawa cells with or without metapristone treatment were measured by real-time PCR (n = 6 per group). Values are means ± SD. **e** The protein expression of *Klf5* and *Nrf1* in the tumor xenograft of RL95-2 cells and Ishikawa cells with or without metapristone treatment were measured by western blot (n = 6 per group). Values are means ± SD. **f** The schematic representation of metapristone inhibitory effect on endometrial cancer cells through regulating cell proliferation and apoptosis and miR-492/*Klf5*/*Nrf1* axis

Previous data suggested us that metapristone had a prefect inhibitory effect on the endometrial cancer cell lines RL95-2 cells and Ishikawa cells in vitro. To further investigate the effect of metapristone in vivo, the tumor xenograft model was established. As Fig. 5a shown, the growth of tumors was obviously inhibited when the mice were treated with metapristone in the model of both RL95-2 cells and Ishikawa cells. Then, the expression of Ki-67 that was a marker of cell proliferation was properly decreased by metapristone, on the other hand, the expression of cleaved-caspase 3 was increased by metapristone through IHC assay (Fig. 5b), which confirmed the impact on cell proliferation and apoptosis by metapristone again. Meanwhile, the IHC results of tumor tissues also demonstrated that *Klf5* and *Nrf1* expression were down-regulated with metapristone treatment (Fig. 5c). Moreover, the mRNA and protein of both RL95-2 cells and Ishikawa cells tumor tissues were extracted. The mRNA expression level of miR-492 was reduced by metapristone in tumor tissues (Fig. 5d). Furthermore, both the mRNA expression (Fig. 5d) and protein expression (Fig. 5e) of *Klf5* and *Nrf1* were lower with the treatment of metapristone than the control groups in vivo. Taken together, all the results showed that metapristone inhibited the endometrial cancer cell growth in vitro and in vivo through regulating miR-492/*Klf5*/*Nrf1* axis and the cell apoptosis-related signaling pathways (Fig. 5f).

## Discussion

Considering that endometrial cancer is a kind of hormonal disorder-mediated gynecological cancers, we chose a hormone-related drug, metapristone, to treat endometrial cancer. Our results presented the noteworthy tumor inhibition effect of metapristone on endometrial cancer in vitro and in vivo through inhibiting cell proliferation and promoting cell apoptosis. However, more in-depth and specific mechanism of metapristone on endometrial cancer is helpful for the clinical treatment. Therefore, this study has focused on the mechanism of metapristone on endometrial cancer to provide the theoretical basis for the clinical treatment.

Many studies provided strong evidence that metapristone was one of mifepristone metabolites with oral administration in mammals and its metabolic pathway was through blood circulation [34, 35], and metapristone had lower cytotoxicity in the systematic monitoring than mifepristone, which was more suitable for the treatment of metastatic cancer [36]. Our results also showed that metapristone maintained more biosecurity in vitro and in vivo (data not shown) compared to mifepristone, which was instructive for its application. Since the tumorigenesis and development of most gender-related cancers have been associated with sex hormones, mifepristone or metapristone has also been clinically applicable to most hormone-related diseases, including contraception and breast cancer [33]. Most of known mechanisms are associated with estrogen, androgen or their receptors. With the enrichment of research, some new mechanisms of metapristone on different types of cancers have been gradually clarified. In particular, metapristone affected ovarian cancer metastasis through interrupting CXCL12/CXCR4 axis [37]. Metapristone suppressed non-small cell lung cancer (NSCLC) cell growth and metastasis by targeting epidermal growth factor receptor (EGFR)-mediated PI3K/AKT pathway [38] and modulating RAS/RAF/MEK/MAPK pathway [39]. Compared to these studies targeting the direct regulation effect of metapristone on cell signaling pathways, our research aimed to find out the new molecular mechanism about the regulation of transcriptional level that was related with the function of miRNAs. There is plenty of evidence to suggest that miRNAs regulate various biological processes in tumorigenesis, development and metastasis of different kinds of cancers [40]. Although miRNAs expression is abnormal in the tumor development and metastasis, the specific function of individual miRNA has been still under investigation. The functional role of miR-492 has been reported in few types of cancers [41–43], such as cervical squamous cell carcinomas [44], hepatoblastoma [45] and colon cancer cells [46]. In this study, we investigated the functional impact of miR-492 on endometrial cancer in vitro and in vivo. Our results firstly indicated that miR-492 was specifically highly-expressed in endometrial cancer cell lines, which suggested that miR-492 was essential for the development of endometrial cancer. The further study demonstrated that *Klf5* and *Nrf1* as the transcription factors were the new targets of miR-492. *Klf5* (also known as BTEB2 and IKLF), a member of the Kruppel-like family of transcription factors, has been implicated as an oncogene and therapeutic target in a number of cancers, including breast, colon, bladder, lung, stomach, and ovarian cancer [47]. The specific biological function of *Klf5* is considered as the transcription factor to regulate cell proliferation, apoptosis, migration, and differentiation [48, 49]. The other study indicated that *Nrf1* was involved in the melanoma carcinogenesis [50]. Our results provided a new research direction for the research of *Klf5* and *Nrf1*. Both of them regulated endometrial cancer cell growth through miR-492. These results also strengthened the understanding of the role of miR-492 in endometrial cancer progression and indicated its diagnostic and prognostic relevance. More importantly, this study suggested that metapristone inhibited endometrial cancer cell growth via miR-492/*Klf5*/*Nrf1* axis, which provided a new mechanism of antitumor effect of metapristone on endometrial cancer for the clinical application.

However, some limitations of this study need to be further broken through. Firstly, some other miRNAs were detected in endometrial cancer cells (Additional Fig. 2) and their expression were lower than miR-492, but a more comprehensive databases validation is still needed to confirm other meaningful miRNAs, which is helpful to find new targeted miRNAs in endometrial cancer. Secondly, the mechanical relationship between *Klf5*/*Nrf1* and cell proliferation or apoptosis with the treatment of metapristone on endometrial cancer has still indistinct. More experiments should be designed to explore the mechanical relationship. In addition, the establishment of tumor mice model can be preferred to some spontaneous endometrial cancer models or PDX tumor models, which is more in line with the actual tumorigenesis and development of endometrial cancer in clinic. Finally, some clinical specimens of endometrial cancer may also be used for further study to verify the function of miR-492 in vivo.

## Conclusion

Taken together, our research indicated that metapristone inhibited endometrial cancer cell growth through suppressing cell proliferation and activating cell apoptosis-related signaling pathway. Mechanically, metapristone regulated miR-492 and its new target genes *Klf5* and *Nrf1 *in vitro and in vivo to treat endometrial cancer. All these results provided a new horizon for treatment of endometrial cancer.

## Supplementary Information

**Additional file 1: Figure 1.** The IC50 concentration of metapristone for different cell lines. **Figure 2.** The different miRNAs relative expression in RL95-2 cells and ISK cells **Table 1.** The sequences of miR-492, Si-miR-492, Nrf1, Klf5 and GAPDH.

## Data Availability

The data used in the current study are available from the corresponding author on reasonable request.

## Abbreviations

CRCs: Colorectal cancers
PCOS: Polycystic ovary syndrome
miRNAs: MicroRNAs
SIX1: *sine oculis* Homeobox homolog 1
ER: Estrogen receptor
PR: Progesterone receptor
AR: Androgen receptor
GR: Glucocorticoid receptor
DMEM: Dulbecco’s modified Eagle’s medium
NCBI: National Center for Biotechnology Information
siRNA: Small interfering RNA
MTT: 3-(4,5-Dimethylthiazol-2-yl)-2,5-diphenyltetrazolium bromide
PBS: Phosphate-buffered saline
qRT-PCR: Quantitative real-time polymerase chain reaction
NSCLC: Non-small cell lung cancer
EGFR: Epidermal growth factor receptor

## Availability of data and materials

The data supporting the conclusions of this study are included within the article.
