# Supplementary material for: Metapristone (RU486-derivative) inhibits endometrial cancer cell progress through regulating miR-492/Klf5/Nrf1 axis
Source: Cancer Cell Int. 2021 Jan 7;21:29. doi: 10.1186/s12935-020-01682-1 (PMC7792070; doi:10.1186/s12935-020-01682-1)
Supplement: Supplementary file 1 — Additional file 1: Figure 1. The IC50 concentration of metapristone for different cell lines. Figure 2. The different miRNAs relative expression in RL95-2 cells and ISK cells Table 1. The sequences of miR-492, Si-miR-492, Nrf1, Klf5 and GAPDH. [file 12935_2020_1682_MOESM1_ESM.docx]

| miR-492 forward | GGCTATGCTTGAGTACG |
| --- | --- |
| miR-492 reverse | CTGAGTTAGCGTACGAGT |
| Si-miR-492 | AAGAAUCUUGUCCCGCAGGUCCU |
| Si-miR-492-NC | CAGUACUUUUGUGUAGUACAA |
| Nrf1 forward | GGACAGCAAGCGATTGTACT |
| Nrf1 reverse | TCTTGTACTTTCGCACCACA |
| Klf5 forward | ACACCAGACCGCAGCTCCA |
| Klf5 reverse | TCCATTGCTGCTGTCTGATTTGTAG |
| GAPDH forward | CTCGCCGCAGTGCATTCGT |
| GAPDH reverse | ACGCTTCGCGATCGTGCGTGAT |

**Additional Table 1:**

**Additional Table 1: The sequences of miR-492, Si-miR-492, Nrf1, Klf5 and GAPDH**.

**Additional Figure 1**


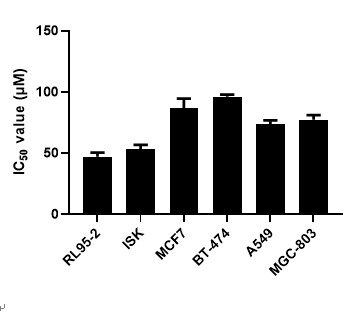


**Additional Fig. 1 The IC_50_ concentration of metapristone for different cell lines**

The IC_50_ values in different cell lines were detected with the treatment of metapristone for 24h. The MTT assay was used to determine the cell viability with the treatment of metapristone for 24h and IC_50_ values were calculated from MTT results.

**Additional Figure 2**


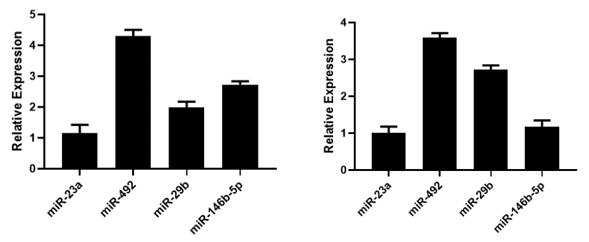


**Additional Fig. 2 The different miRNAs relative expression in RL95-2 cells and ISK cells.**

The relative expression of miR-23a, miR-492, miR-29b and miR-146b-5p were detected by real-time PCR. The left is the results of RL95-2 cells and the right is the results of ISK cells.
